# Supplementary material for: Parkin deficiency rewires the genetic dependencies that govern neuronal survival
Source: bioRxiv. 2026 Oct 28:2026.10.27.762340. Preprint. [Version 1] doi: 10.64898/2026.10.27.762340 (PMC13733959; doi:10.64898/2026.10.27.762340)
Supplement: Supplementary file 1 [file NIHPP2026.10.27.762340V1-supplement-1.pdf]

bioRxiv preprint doi: https://doi.org/10.64898/2026.10.27.762340; this version posted October 28, 2026. The copyright holder for this preprint (which was not certified by peer review) is the author/funder, who has granted bioRxiv a license to display the preprint in perpetuity. It is made available under a CC-BY 4.0 International license.

Supplementary Materials

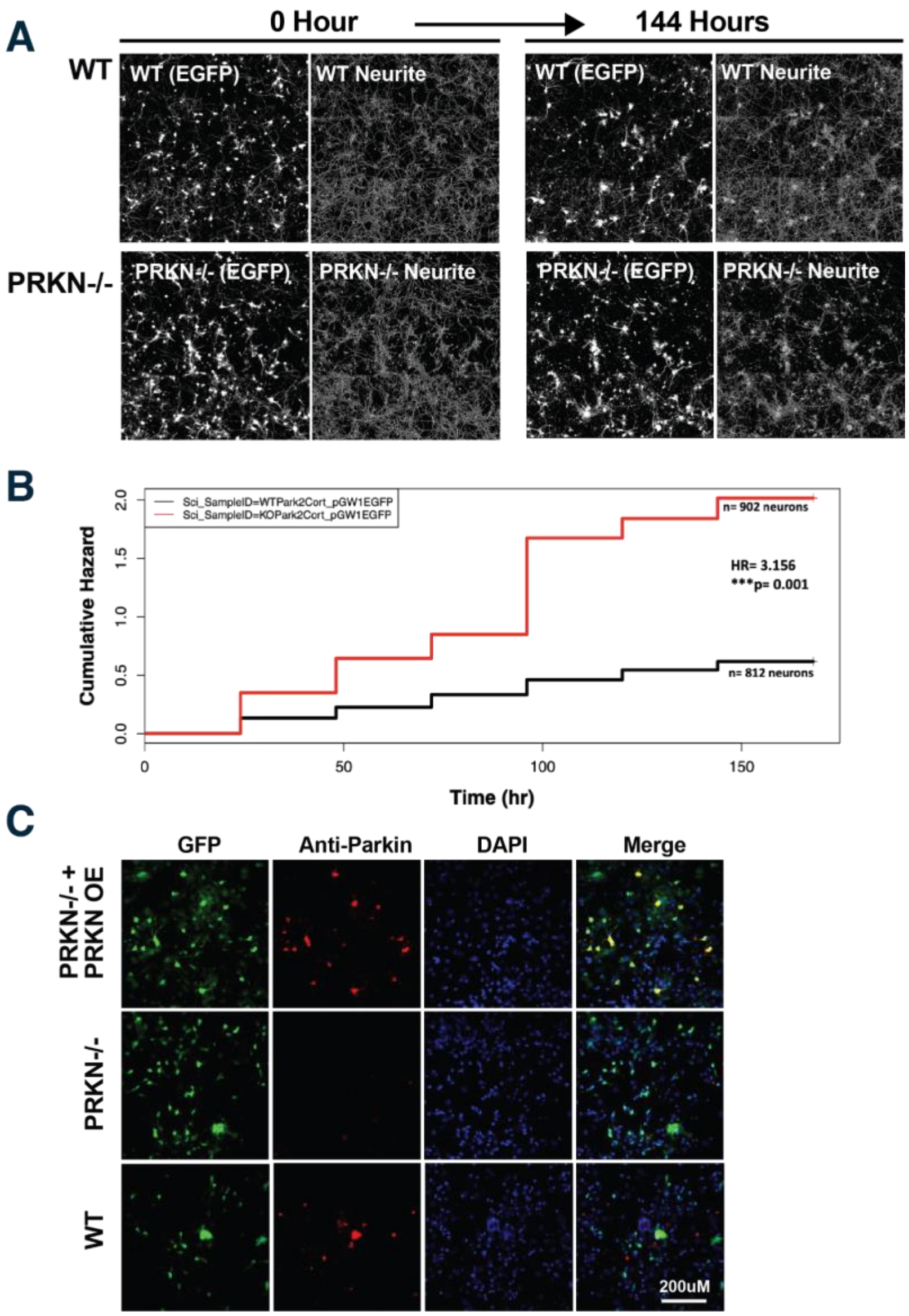

Supplementary Figure 1. Extended characterization of neuronal survival and mitochondrial phenotype in Parkin-deficient neurons.

A) Representative fluorescence images showing neuronal morphology (EGFP channel) and neurite architecture (Neurite channel) in WT and PRKN$^{-/-}$ neurons used for manual longitudinal tracking. **B)** Manual longitudinal survival tracking of more than 800 individual neurons across two independent experiments over seven days (red step-function curve). PRKN$^{-/-}$ neurons show a 3.1-fold higher death rate than WT controls (HR = 3.1, $p < 0.001$), consistent with the automated GEDI-based result in Figure 1C. **C)** Representative fluorescence images showing immunostaining of Parkin in WT, Parkin$^{-/-}$ and Parkin$^{-/-}$ with Parkin overexpression (Parkin$^{-/-}$ + Parkin OE) neurons. Scale bar = 200 μm.

bioRxiv preprint doi: https://doi.org/10.64898/2026.10.27.762340; this version posted October 28, 2026. The copyright holder for this preprint (which was not certified by peer review) is the author/funder, who has granted bioRxiv a license to display the preprint in perpetuity. It is made available under a CC-BY 4.0 International license.

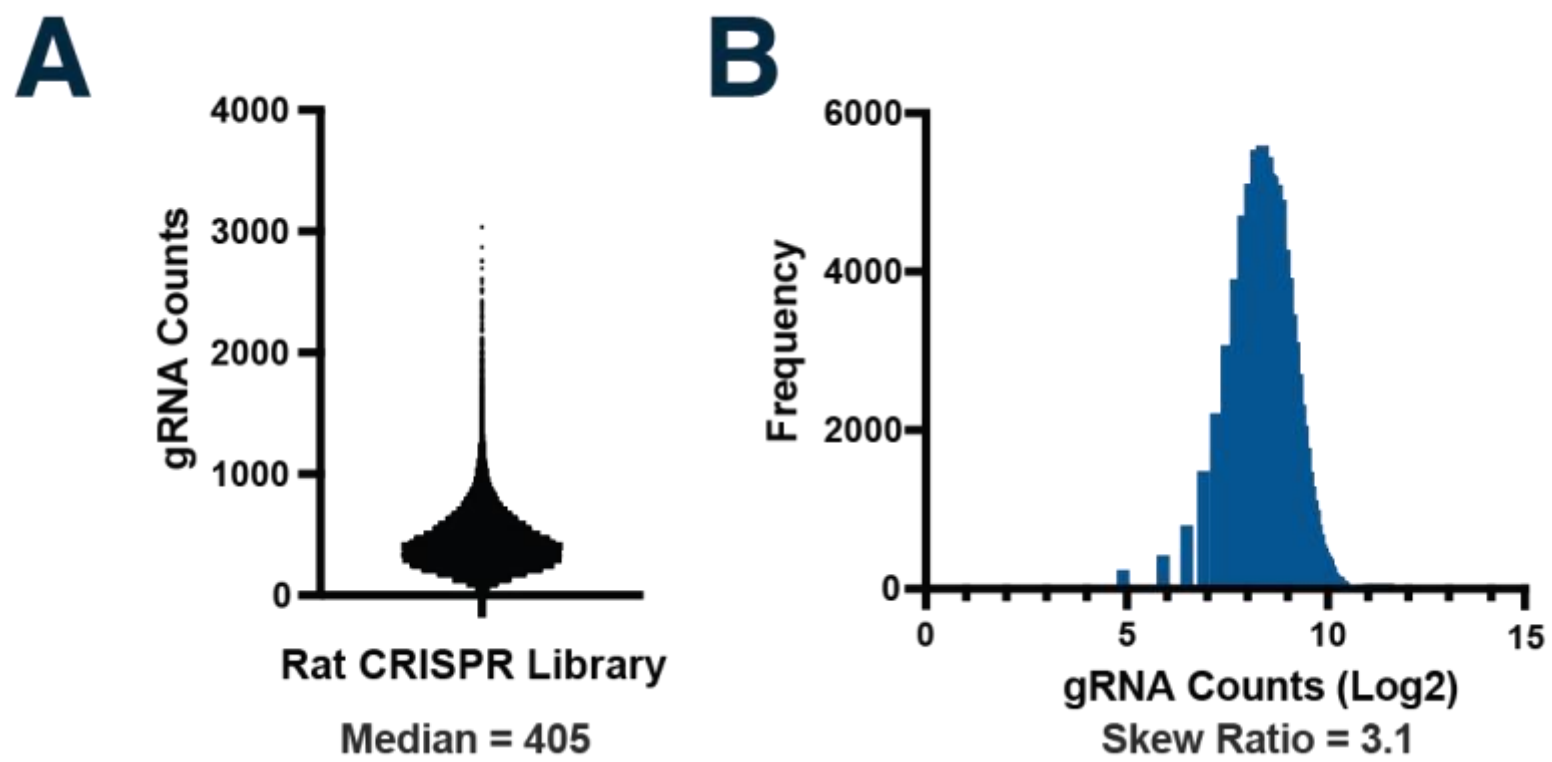

Supplementary Figure 2. Quality control of the rat neuronal CRISPR library.

A) Violin plot of raw sgRNA counts in the final rat CRISPR library. Each point represents one sgRNA; the violin shape reflects the count distribution across all 87,329 sgRNAs. Median count = 405, indicating adequate sequencing depth. **B)** Histogram of log_2-transformed sgRNA counts. The distribution is unimodal and approximately symmetric with a skew ratio = 3.1, confirming balanced library representation.

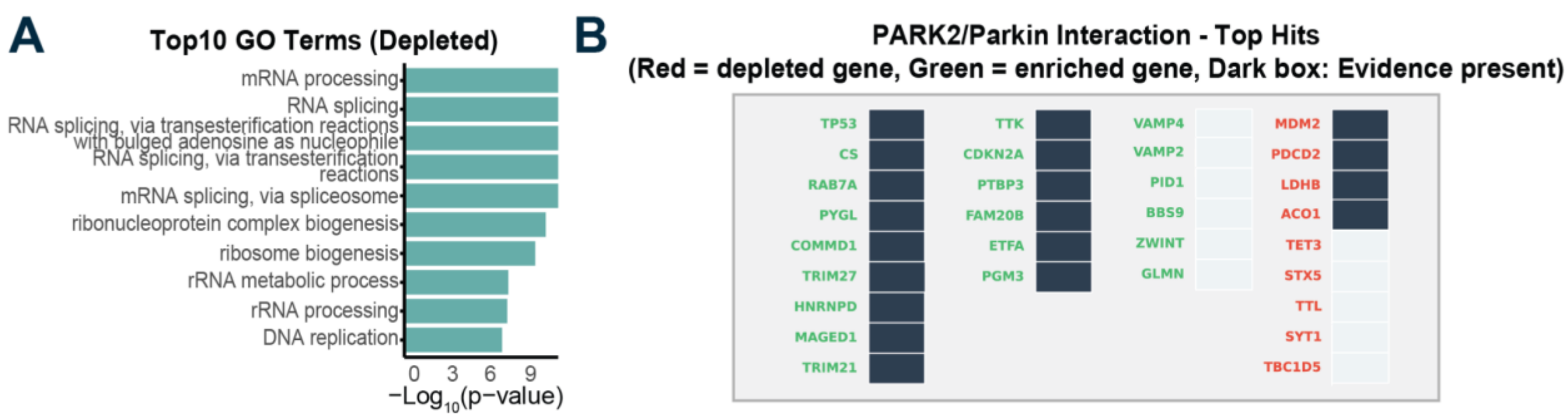

Supplementary Figure 3. Pathway enrichment of depleted screen hits and Parkin interaction analysis of CRISPR screen candidates.

A) Gene Ontology over-representation analysis of the full set of negatively enriched genes before removal of pan-essential genes. The top 10 enriched biological process terms include mRNA processing, RNA splicing, ribonucleoprotein complex biogenesis, and ribosome biogenesis, reflecting the expected recovery of housekeeping essential genes in a depletion screen. These pathways were excluded from the primary hit analysis by cross-referencing DepMap CRISPR gene effect scores in CNS and peripheral nervous system cell lines. **B)** Grid display of CRISPR screen hits with confirmed physical interactions with PARK2/Parkin in BioGRID. Green gene names = enriched in screen; red gene names = depleted in screen. Dark boxes indicate direct biochemical evidence for the Parkin interaction (supported by low-throughput, direct biochemical assays (Biochemical Activity, Reconstituted Complex, or Affinity Capture). White boxes indicate high-throughput interactions were those identified exclusively in large-scale proteomics screens.

bioRxiv preprint doi: https://doi.org/10.64898/2026.10.27.762340; this version posted October 28, 2026. The copyright holder for this preprint (which was not certified by peer review) is the author/funder, who has granted bioRxiv a license to display the preprint in perpetuity. It is made available under a CC-BY 4.0 International license.

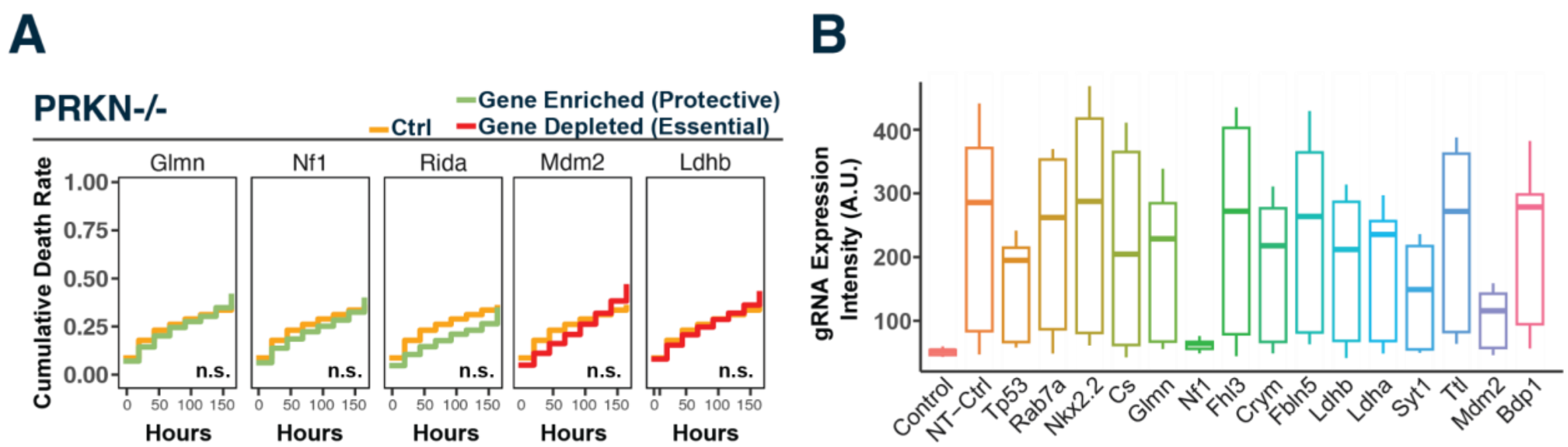

Supplementary Figure 4. Validation of non-replicating arrayed gene knockouts and CRISPR-gRNA expression

A) Cumulative death curves in PRKN$^{-/-}$ neurons for the five candidate genes that showed trends in the expected direction but did not reach statistical significance in the arrayed format: *Glmn, Nf1, Rida, Mdm2, and Ldhb* (all n.s.; *Rida*: HR = 0.78, adj. p = 0.0502; *Ldhb*: HR = 1.15, adj. p = 0.40; *Mdm2*: HR = 1.15, adj. p = 0.40; *Glmn*: HR = 1.09, adj. p > 0.99; *Nf1*: HR = 1.00, adj. p > 0.99). **B)** Box plot of gRNA expression intensity (arbitrary units) for each gene targeted in the arrayed validation screen, measured by fluorescence imaging of the GFP-linked sgRNA reporter. Most targeted genes show detectable CRISPR-gRNA expression above the NT-Ctrl baseline, confirming successful lentiviral delivery across conditions.

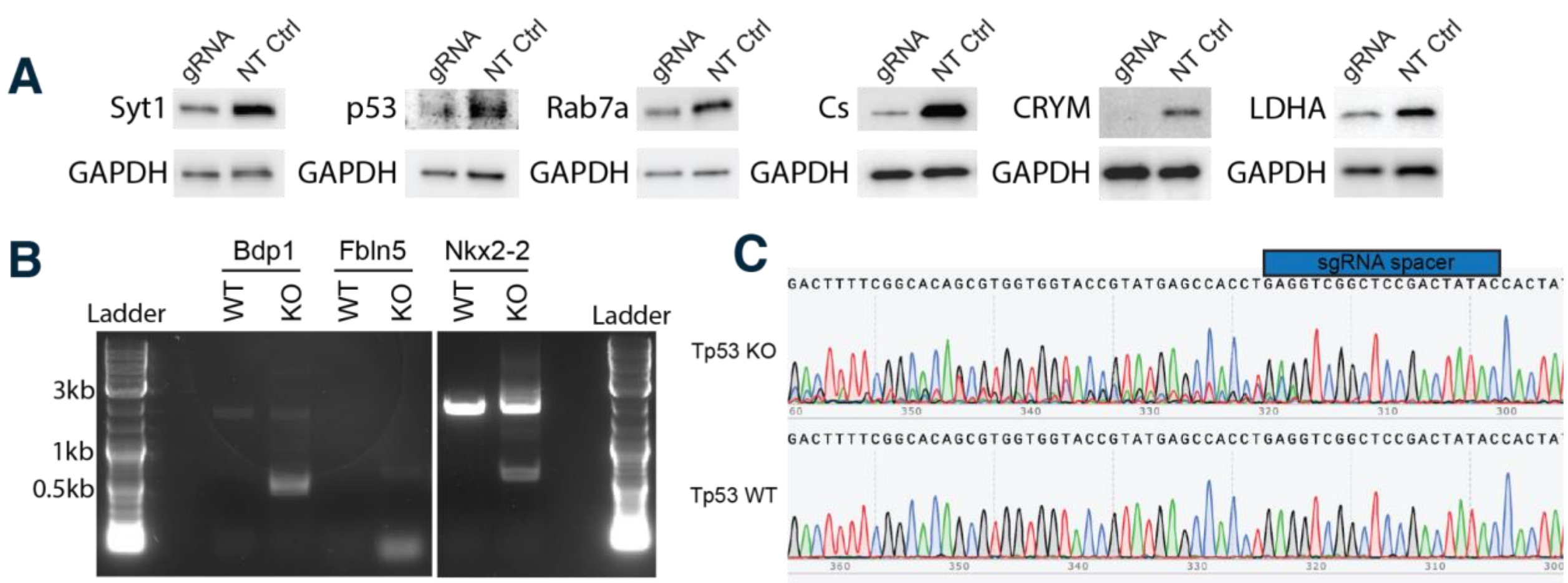

Supplementary Figure 5. Validation of replicating arrayed **gene knockouts**

A) Western blot validation of protein knockdown for six genes targeted in the arrayed screen: Syt1, p53, Rab7a, Cs, Crym, and Ldha. GAPDH serves as a loading control. Target protein levels are reduced in CRISPR-gRNA expressing neurons relative to non-targeted controls, confirming successful gene knockout at the protein level. **B)** PCR gel showing genomic editing at *Bdp1*, *Fbln5*, and *Nkx2-2* loci in WT and PRKN$^{-/-}$ neurons. Shifted or absent bands in the PRKN$^{-/-}$ lanes confirm successful Cas9-mediated disruption of each target locus. **C)** Sanger sequencing traces at the *Tp53* locus confirming CRISPR editing. The *Tp53* KO trace (top) shows mixed/noisy sequence downstream of the cut site, indicating indel formation; the *Tp53* WT trace (bottom) shows a clean, unperturbed sequence at the same position.
